# Prophylactic Peripheral Blood Stem Cell Collection in Patients with Extensive Bone-Marrow Infiltration of Neuroendocrine Tumours Prior to Peptide Receptor Radionuclide Therapy with ^177^Lu-DOTATATE

**DOI:** 10.3390/ph14101022

**Published:** 2021-10-05

**Authors:** Amir Sabet, Nicolai Mader, Jörg Thomas Bittenbring, Fadi Khreish, Frank Grünwald, Hans Jürgen Biersack, Samer Ezziddin

**Affiliations:** 1 Department of Nuclear Medicine, University Hospital Frankfurt, 60590 Frankfurt am Main, Germany; nicolai.mader@kgu.de (N.M.); frank.gruenwald@kgu.de (F.G.); 2 Department of Haematology and Oncology, Caritas Hospital Saarbrücken, 66123 Saarbrücken, Germany; Joerg.Thomas.Bittenbring@uks.eu; 3 Department of Nuclear Medicine, Saarland University Medical Center, 66421 Homburg, Germany; fadi.khreish@uks.eu (F.K.); samer.ezziddin@uks.eu (S.E.); 4 Department of Nuclear Medicine, University Hospital Bonn, 53127 Bonn, Germany; hans-juergen.biersack@betaklinik.de

**Keywords:** peptide receptor radionuclide therapy, PRRT, neuroendocrine tumor, NET, peripheral blood stem cell collection, PBSC, hematotoxicity

## Abstract

Peptide receptor radionuclide therapy (PRRT) of metastatic neuroendocrine tumors (NET) can be successfully repeated but may eventually be dose-limited. Since ^177^Lu-DOTATATE dose limitation may come from hematological rather than renal function, hematological peripheral blood stem cell backup might be desirable. Here, we report our initial experience of peripheral blood stem-cell collection (PBSC) in patients with treatment-related cytopenia and therefore high risk of bone-marrow failure. Five patients with diffuse bone-marrow infiltration of NET and relevant myelosuppression (≥grade 2) received PBSC before one PRRT cycle with ^177^Lu-DOTATATE (7.6 ± 0.8 GBq/cycle). Standard stem-cell mobilization with Granulocyte-colony stimulating factor (G-CSF) was applied, and successful PBSC was defined as a collection of >2 × 10^6^/kg CD34+ cells. In case of initial failure, Plerixafor was administered in addition to G-CSF prior to apheresis. PBSC was successfully performed in all patients with no adverse events. Median cumulative activity was 44.8 GBq (range, 21.3–62.4). Three patients had been previously treated with PRRT, two of which needed the addition of Plerixafor for stem-cell mobilization. Only one of five patients required autologous peripheral blood stem-cell transplantation during the median follow up time of 28 months. PBSC collection seems to be feasible in NET with bone-marrow involvement and might be worth considering as a backup strategy prior to PRRT, in order to overcome dose-limiting bone-marrow toxicity.

## 1. Introduction

Peptide receptor radionuclide therapy (PRRT) with ^177^Lu-DOTATATE is a longstanding systemic treatment for metastatic gastroenteropancreatic neuroendocrine tumors (GEP- NET) [1,2,3]. Over recent years, several studies have reported remarkable results of individualized PRRT schemes for selected patients in different settings [4,5,6,7,8,9,10,11,12,13,14]. However, the only conducted randomized phase III trial (NETTER-1) demonstrating the efficacy of PRRT was limited to patients with midgut NET receiving a “standard treatment scheme” consisting of four cycles with a predefined administered activity of 7.4 GBq per cycle every 8 weeks [15]. Therefore, identifying other eligible patients for PRRT, appropriate treatment individualizations, and baseline characteristics influencing the outcome mainly relies on data from retrospective studies [16,17,18,19,20]. To date, ki67-index <20% and limited liver tumor burden are considered as the main favorable prognostic factors for NET patients undergoing PRRT, irrespective of the primary tumor [8,21,22]. Patients with advanced metastatic bone involvement, on the other hand, are at higher risk of bone-marrow impairment and are treated cautiously to avoid severe irreversible myelotoxicity [23,24,25]. In view of the low overall toxicity of PRRT, especially the low nephrotoxic potential of ^177^Lu-DOTATATE, myelosuppression may also be the cumulative dose-limiting toxicity in responsive patients, restricting the application of repeat PRRT cycles [2,26,27,28].

Autologous peripheral blood stem-cell transplantation is widely used after myeloablative treatments in a variety of hematological malignancies and solid tumors. Peripheral blood is the usual source of stem cells, providing a safe and easily accessible route for isolating autologous stem cells [29,30]. Different strategies have been applied to optimize the mobilization and collection of peripheral blood stem cells (PBSC), especially in patients with risk factors for poor mobilization such as bone-marrow involvement [31,32]. To the best of our knowledge, this concept has not yet been considered for PRRT and it remains unclear whether PBSC can be collected in bone metastatic NET disease. If proven successful and safe, autologous transplantation of PBSC may encourage the use of higher activities and more cycles, especially in patients with advanced metastatic disease and very few therapeutic options, increasing the clinical benefit and expanding the indications for PRRT. In this paper we introduce and discuss the feasibility of PBSC collection in NET patients with metastatic bone-marrow spread.

## 2. Methods

Five patients with bone-marrow infiltration of NET and cytopenia on complete blood counts (≥grade 2 according to CTCAE v5.0) received PBSC apheresis 8 ± 4 weeks before the onset (*n* = 2) or in the interval of two ^177^Lu-DOTATATE administrations (*n* = 3). PRRT was performed with 7.6 ± 0.8 GBq ^177^Lu-DOTATATE per cycle at standard intervals of 3 months using a previously described protocol [22]. Prior to PBSC, standard stem-cell mobilizing agent granulocyte-colony stimulating factor (G-CSF) was applied subcutaneously at 10 µg/kg/day for at least 4 days to achieve CD34+ cell counts ≥ 10/µL as the prerequisite for the apheresis. Successful PBSC was defined as >2 × 10^6^/kg CD34+ cells [31]. If adequate numbers of stem cells could not be mobilized, the selective CXCR4 antagonist Plerixafor was added to G-CSF to enhance the outcome (2,40 mg/kg s.c.,10 h before apheresis) [32,33]. Table 1 provides tumor- and treatment-related characteristics of the patient cohort.

## 3. Results

**Figure 1:**
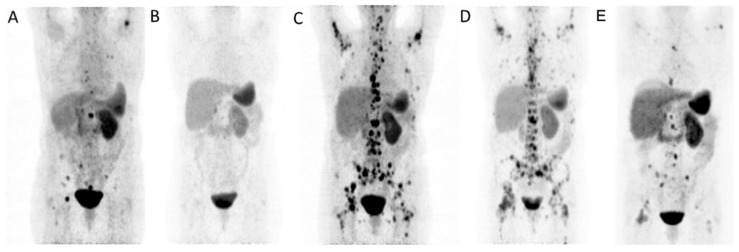
Maximum-intensity-projection ^68^Ga-DOTATOC PET images of a patient with renal NET before (**A**) and after (**B**) four cycles of ^177^Lu-DOTATATE (29 GBq) showing initial response. Repeat PRRT was performed 14 months later because of progressive disease (**C**) after successful PBSC harvesting and almost achieved disease stabilization (**D**) after 2 cycles (15.4 GBq) of re-PRRT and partial response (**E**) after additional 3 cycles (18 GBq).

All patients had relevant cytopenia (≥grade II) of at least one blood cell line at the time of apheresis. Three patients had anemia (two grade II, and one grade III), two patients had thrombocytopenia (one grade III, and one grade IV), and three patients had leukopenia (one grade II, and two grade III). The median cumulative activity of ^177^Lu-DOTATATE was 44.8 GBq (range, 21.3–62.4; mean, 42.1). PBSC was successfully performed in all five patients. Two of three patients with progressive disease under chemotherapy showed relevant cytopenia at baseline and received PBSC prior to initiation of PRRT, adequate mobilization was achieved in both with G-CSF only. In contrast, two of three patients previously treated with PRRT required additional Plerixafor to achieve >2 × 10^6^/kg CD34+ cells. During the mean follow-up of 28 months, one patient required autologous stem-cell support because of severe cytopenia (thrombocytopenia, grade IV; neutropenia, grade III) 70 days after the completion of PRRT. Blood counts recovered to near normal (grade I thrombocytopenia, normal neutrophil and leukocyte counts) 6 weeks after the procedure. In the other four patients, the harvested PBSC remained stored as back-up material in case of future bone-marrow failure. Figure 1 illustrates 68Ga-DOTATOC PET images of a patient with renal NET treated with 29 GBq 177Lu-DOTATATE prior to PBSC harvesting.

## 4. Discussion

This preliminary report introduces the concept of PBSC in high-risk patients with advanced bone-marrow involvement of NET undergoing PRRT, describing a successful procedure in five patients with bone-marrow impairment at the time of apheresis.

Few therapeutic options exist for patients with advanced stage metastatic NET. PRRT with ^177^Lu-DOTATATE is a potent systemic treatment modality for metastatic NET with a low overall toxicity profile. However, the risk of therapy-induced bone-marrow failure restricts the application of sufficiently high activities in patients with extensive bone-marrow infiltration. Similarly, patients with low or decreasing hematological parameters are generally treated with reduced activities, leading to suboptimal treatment outcomes in patients with high tumor load or a clinical need for tumor remission. Patients with significant cytopenia of ≥grade III are often discarded from PRRT [23,25,34]. For a substantial portion of patients with metastatic NET undergoing PRRT, it may persist as a long-term palliative treatment as it can reinduce tumor response once re-progression occurred [16,35,36,37]. However, cumulative absorbed dose to bone marrow would eventually limit the application [38,39]. Moreover, long survival of NET patients allows for the clinical occurrence of myelodysplastic syndrome, a potential late sequel of alkylating chemotherapy and systemic radionuclide therapies [40].

If proved feasible in clinical practice, autologous transplantation of PBSC may contribute to overcome the dose-limiting hematotoxicity, allowing dose escalations or repeat PRRT cycles in selected patients. This approach has been successfully used in patients with malignant pheochromocytomas and paragangliomas treated with ^131^I-Metaiodobenzylguanidine (^131^I-MIBG). In a phase II study by Fitzgerald et al., PBSC collection at baseline enabled an individualized high-dose treatment with a mean of 833 mCi (557–1185 mCi) in 30 patients. In 4/30 patients, bone-marrow support was needed after the treatment and effectively prevented irreversible myelosuppression [41]. Similarly, in our series, blood counts of the patient with severe cytopenia recovered after autologous peripheral blood stem-cell transplantation. 

Hematopoietic growth factors like G-CSF are used for releasing stem cells from the bone marrow into the peripheral blood. The selective CXCR4 antagonist AMD3100 (Plerixafor) [42,43], has been proven to enhance PBSC collection in cancer patients and is established for this purpose, especially in case of mobilization issues [33]. Plerixafor was used in two of our patients to achieve sufficient cell mobilization. Therefore, the most appropriate time point for prophylactic PBSC remains to be determined. Although PBSC would possibly be easier prior to initiation of PRRT [32], collection at later time points (e.g., after two cycles) may facilitate the assessment of risk for developing bone-marrow failure under therapy. In our small cohort, successful PBSC was possible in all patients even after high cumulative activities of ^177^Lu-DOTATATE.

## 5. Conclusions

Prophylactic PBSC seems technically feasible in patients with advanced bone metastatic NET, even after PRRT with high cumulative activity. The procedure might be worth considering especially in high-risk candidates in an attempt to overcome the dose-limiting hematological toxicity of PRRT.

## Figures and Tables

**Table 1:** Patients’ characteristics and details of PBSC.

Age/Sex	Prmary/Grade	Previous Therapies	Metastatic Site	Mobilizing Agent	PBSC Results	Activity (Cycles)		Response
						Before PBSC	After PBSC	
**60y/w**	Ileum/G2	CTx (Dox/5FU), SSA	liver, bone, LN	G-CSF	Successful	0 GBq (0)	43.0 GBq (6)	SD
**54y/w**	Kidney/G2	Surgery, SSA, PRRT	bone	G-CSF	Successful	29.0 GBq (4)	33.4 GBq (5)	PR
**70y/m**	Jejunum/G1	Surgery, SSA, PRRT	liver, bone	G-CSF, Plerixafor	Successful	15.7 GBq (2)	5.6 GBq (1)	PD
**53y/m**	CUP/G2	CTx (Cis/5FU), Radiation	liver, bone, LN	G-CSF	Successful	0 GBq (0)	39.0 GBq (6)	PR
**58y/w**	Pancreas/G2	CTx (STZ/5FU), SSA Surgery, PRRT	liver, bone	G-CSF, Plerixafor	Successful	37.3 GBq (5)	7.5 GBq (1)	PR

CUP, cancer of unknown origin; SSA, somatostatin receptor analogues; CTx, chemotherapy; STZ, streptozotocin; Dox, doxorubicin; Cis, cisplatin; 5FU, 5-fluorouracil; PRRT, peptide receptor radionuclide therapy.

## Data Availability

Data is contained within the article.

## Abbreviations

^177^Lu	Lutetium-177
CTCAE	Common Terminology Criteria for Adverse Events
G-CSF	Granulocyte-colony stimulating factor
NET	Neuroendocrine tumor
PBSC	Peripheral blood stem-cells collection
PRRT	Peptide receptor radionuclide therapy
